# Healthcare-associated respiratory viral infections after discontinuing universal masking

**DOI:** 10.1017/ice.2023.200

**Published:** 2024-02

**Authors:** Zachary M. Most, Bethany Phillips, Michael E. Sebert

**Affiliations:** 1 Division of Infectious Disease, Department of Pediatrics, University of Texas Southwestern Medical Center, Dallas, Texas; 2 Children’s Health System of Texas, Infection Prevention and Control, Dallas, Texas

## Abstract

In November 2022, our pediatric hospital replaced the requirement for universal masking of all healthcare personnel and visitors in all clinical buildings with a requirement for masking only during patient encounters. Following this change, we observed an immediate, substantial, and sustained increase in healthcare-associated respiratory viral infections.

During the coronavirus disease 2019 (COVID-19) pandemic, universal masking of all healthcare personnel (HCP) and visitors was implemented in most hospitals in the United States to mitigate transmission of severe acute respiratory syndrome coronavirus 2 (SARS-CoV-2). Like other hospitals, we observed a concurrent and rapid decline in the incidence of healthcare-associated respiratory viral infections (HARVIs).^1–3^ Universal masking was implemented simultaneously with a large reduction in community respiratory viral infection incidence and numerous mitigation strategies in healthcare settings and the community, which also may have affected HARVI rates. These included universal hospital admission and preprocedural testing for SARS-CoV-2 that often also identified other viruses, hospital visitor restrictions, increased use of personal protective equipment and hand hygiene, and physical distancing. Therefore, lower HARVI rates may not have been solely due to universal masking. In our pediatric hospital, the requirement for universal masking in all clinical buildings was discontinued on November 8, 2022. No other COVID-19–related mitigation strategies changed at that time, including visitor restrictions and HCP work exclusions (see Supplementary Background Material online). This situation provided an opportunity to evaluate the effectiveness of universal masking in healthcare settings in preventing HARVIs.

## Methods

Throughout most of the COVID-19 pandemic, our 490-bed pediatric hospital had required masking by HCPs, patients, and visitors in all clinical buildings. On November 8, 2022, this mandate was replaced with a narrower requirement that all HCP wear a medical mask (or higher-grade respirator if indicated) when providing direct patient care and encouraged patients and families to mask for these same episodes. A definite HARVI was defined as an infection meeting 3 criteria: (1) microbiologic criterion including an upper or lower respiratory specimen testing positive on an antigen test or respiratory pathogen multiplex polymerase chain reaction panel for adenovirus, endemic human coronavirus (HKU1, NL63, 229E, or OC43), human metapneumovirus, influenza A (H1N1pdm2009 or H3N2), influenza B, human parainfluenza virus (type 1, 2, 3, or 4), respiratory syncytial virus (RSV), rhinovirus and enterovirus (which are not distinguished on the panel), or SARS-CoV-2; (2) symptomatic criterion including at least 1 new sign or symptom associated with a lower or upper respiratory infection following hospital admission; and (3) chronologic criterion including the onset of symptoms was on or after a minimum number of days from hospital admission specific for each virus’s maximum incubation period (see Supplementary Methods online).^4^

To analyze monthly HARVI rates, we used a statistical process control *u* chart (implemented in QIMacros, KnowWare International, Denver, CO). The average monthly HARVI rate during a baseline period from January 2019 to December 2019 was used to calculate the initial centerline. Monthly data points were marked as out of control according to control chart analysis rules from the Institute for Healthcare Improvement.^5^ A centerline shift was made when there were 8 consecutive months above or below the centerline.

## Results

**Figure 1.**
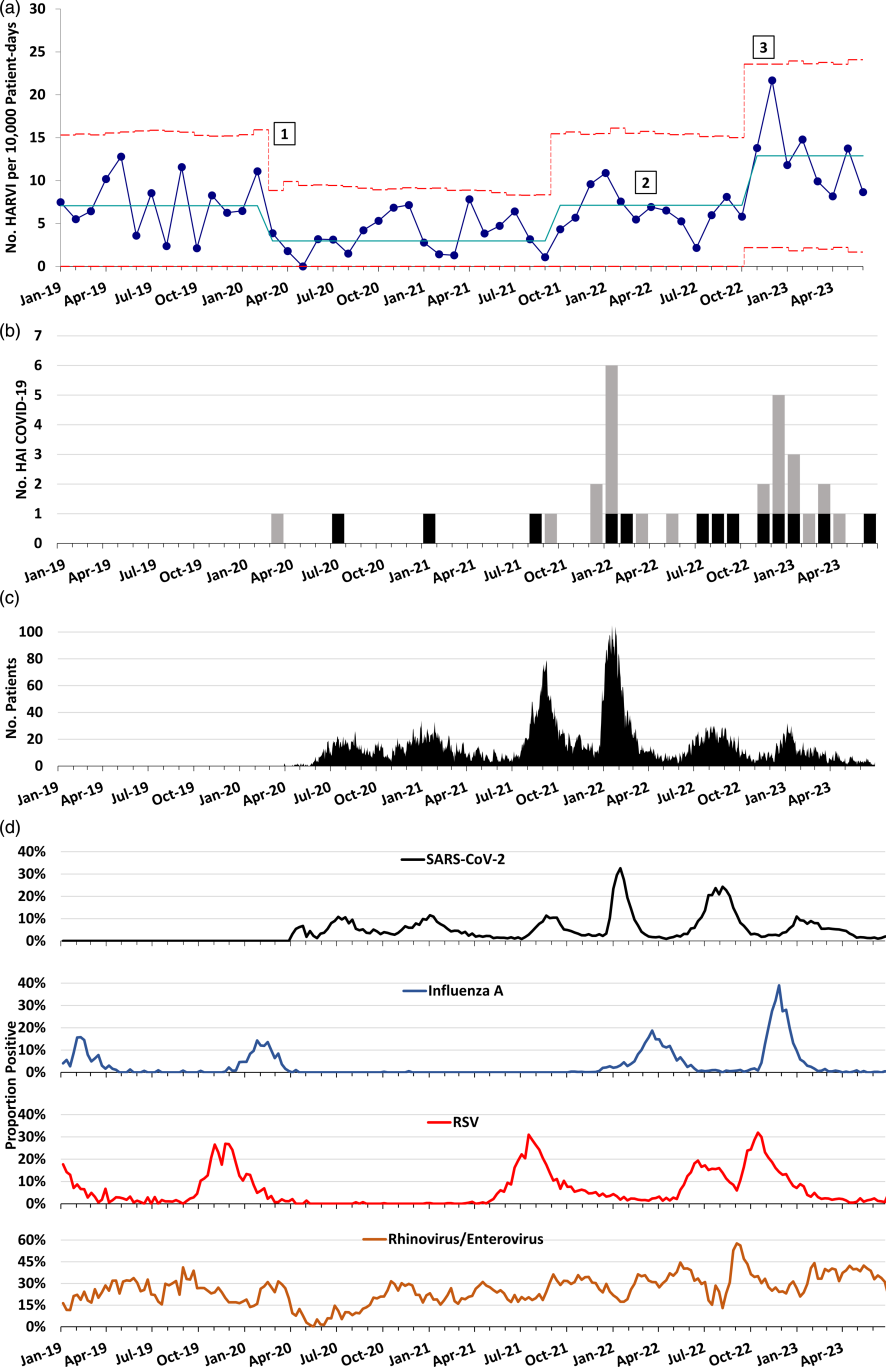
Trend in healthcare-associated and community respiratory viral infections over time at a pediatric hospital. (A) Statistical process control *u* chart showing incidence of healthcare-associated respiratory viral infections (HARVIs) by month. Solid blue line: centerline. Red dashed lines indicate upper control limit (+3 sigma) and lower control limit (−3 sigma). Centerline shifts occurred when there were 8 consecutive months above or below the centerline, according to Institute for Healthcare Improvement control chart rules. Numbers mark (1) initiation of universal masking and SARS-CoV-2 admission testing; (2) discontinuation of SARS-CoV-2 admission testing for asymptomatic individuals with no known coronavirus disease 2019 (COVID-19) close contacts; and (3) replacement of universal masking with masking during all patient encounters. HARVI case definitions are provided in the Supplementary Appendix (online). (B) Run chart of definite (gray bars) and possible (black bars) healthcare-associated COVID-19 cases by month. (C) Hospital census of patients admitted with SARS-CoV-2 infection by date. (D) Test positivity rate for several respiratory viruses in the hospital microbiology laboratory by week.

Prior to the COVID-19 pandemic, our centerline for HARVI incidence was 7.07 infections per 10,000 patient days. At the start of the pandemic, the HARVI incidence decreased, with a centerline shift down to 2.60 infections per 10,000 patient days. However, an increase in HARVI incidence occurred from the late fall of 2021 through spring 2022 that resulted in a centerline shift back to the prepandemic baseline rate of 7.13 infection per 10,000 patient days. This increase coincided with the beginning of the SARS-CoV-2 B.1.1.529 (omicron) variant wave and included relaxation of the hospital visitor restrictions policy and the end of universal admission testing in March and April 2022, respectively. When universal masking was discontinued in November 2022, we observed a sharp rise in HARVIs that exceeded our prepandemic baseline (Fig. 1a). This rise has been sustained for 8 consecutive months with rates above the centerline, resulting in another centerline shift to 12.88 infections per 10,000 patient days. Infections with rhinovirus and/or enterovirus have represented the largest proportion of HARVIs since November 2022, but other viruses including influenza, RSV, and SARS-CoV-2 have contributed to the total (see Supplementary Table 1 online). Time from admission to HARVI was similar throughout the observation period, with median intervals substantially greater than the maximum incubation periods for the viruses (Supplementary Table 2 online). Concurrently, there was a rise in definite or possible healthcare-associated COVID-19 cases from November 2022 through June 2023 (Fig. 1b). This increase occurred despite having a relatively low census of admitted patients with COVID-19 during this interval (Fig. 1c) and despite declining local prevalences of multiple respiratory viral infections including RSV and influenza (Fig. 1d and Supplementary Figs. 1–5 online).

The following viruses caused HARVIs from November 2022 through June 2023: rhinovirus and enterovirus (n = 41), parainfluenza virus (n = 13), adenoviruses (n = 11), SARS-CoV-2 (n = 8), endemic coronaviruses (n = 6), influenza A (n = 4), human metapneumovirus and RSV (n = 3 each), and 13 coinfections (Supplementary Table 1 online). During the same period, the median time from admission to HARVI was 24.5 days (interquartile range, 13–64.75), which was similar to earlier periods (Supplementary Table 2 online).

## Discussion

When our hospital moved from universal masking in all clinical buildings to masking for all patient encounters only, we observed an immediate, substantial, and sustained increase healthcare-associated respiratory viral infections. These observations are informative regarding the impact of masking because—unlike rapid simultaneous implementation of numerous mitigation strategies in healthcare settings and the community early in the pandemic—no other changes in infection prevention strategies or staffing levels were implemented at our facility in late 2022. The elevated transmission pressure from community viruses during the winter respiratory virus season may explain part but not all of the increase in HARVI rate. Cases of healthcare-associated COVID-19 during this period approached what was seen during the omicron wave despite a consistently lower census of admitted patients positive for SARS-CoV-2. Moreover, local prevalences of most common respiratory viruses were steady or declining during the period when the HARVI rate rose dramatically.

We also observed an increase in HARVI incidence between October 2021 and spring 2022. This increase coincided with multiple changes in respiratory virus epidemiology and relaxation of several COVID-19 mitigation strategies in the hospital, so it was more difficult to isolate the effect of any individual intervention. Nonetheless, this earlier observation contributes additional evidence that COVID-19 mitigation strategies were effective in reducing respiratory virus transmission in our hospital.

This study had several limitations. We were unable to ascertain where transmissions may have taken place when masking became optional outside clinical encounters (eg, hallways, elevators, or cafeterias). We only had 8 months of follow-up data. We were unable to control for all time-varying exogenous factors that can be associated with HARVIs. Also, we did not have data on HCP adherence to mask usage. However, the magnitude and close temporal association of the rise in HARVIs with discontinuation of universal masking support an independent impact of universal masking on spread of respiratory viral infections. This increase occurred despite an ongoing requirement for all HCP to wear medical masks for all patient encounters. The increase in HARVI rate to a level above the prepandemic baseline could indicate a drift in HCP and patient visitor adherence to respiratory protection including fatigue with PPE usage and/or increased presenteeism.

In conclusion, our findings suggest that universal masking in our children’s hospital was effective in reducing respiratory virus transmission and that masking only for all patient encounters was not as effective. Universal masking should be considered as part of the infection prevention toolbox in certain scenarios.

## Supplementary material

For supplementary material accompanying this paper visit https://doi.org/10.1017/ice.2023.200. click here to view supplementary material
