# Supplementary material for: Healthcare-associated respiratory viral infections after discontinuing universal masking
Source: Infect Control Hosp Epidemiol. 2023 Sep 25;45(2):247–9. doi: 10.1017/ice.2023.200 (PMC10877529; doi:10.1017/ice.2023.200)

Supplemental Appendix for ‘Healthcare-associated Respiratory Viral Infections After Discontinuing Universal Masking'

Supplemental Background

Additional COVID-19 mitigation strategy changes that occurred in 2022

- In March 2022, hospital visitor restrictions for patients other than those with COVID-19 were lifted. Those with COVID-19 were limited to one of two designated visitors at a time. The neonatal intensive care unit (NICU) implemented seasonal visitor restrictions in late October 2022 due to the onset of respiratory virus season.
- In April 2022, universal admission testing for COVID-19 along with other respiratory viruses was discontinued.
- In September 2022, occupational health work exclusion rules were updated to adhere to changes recommended by the Centers for Disease Control and Prevention (CDC). Prior to those changes, vaccinated HCP were not work restricted following a known exposure to COVID-19 if they were asymptomatic. In September 2022 this was extended to unvaccinated HCP as well. We also increased the number of negative antigen tests required for asymptomatic exposed HCPs (from 2 to 3 per CDC guidelines).

Supplemental Methods

HARVI Definition

A definite HARVI was defined as an infection meeting three criteria:

1. Microbiologic criterion: an upper or lower respiratory specimen testing positive on an antigen test (Quidel Sofia^®^ Influenza A+B FIA, Quidel Sofia^®^ RSV FIA, or Quidel Sofia^®^ Flu+SARS Antigen FIA [Quidel Corporation, San Diego, California]) or a respiratory pathogen multiplex polymerase chain reaction (PCR) panel (BioFire^®^ FilmArray^®^ Respiratory Panel, BioFire^®^ FilmArray^®^ Respiratory Panel 2.1 [bioMérieux, Marcy-l'Étoile, France], Xpert Xpress influenza/RSV PCR, or Xpert Xpress Cov-2/influenza/RSV PCR [GeneXpert^®^ Dx system, Cepheid^®^, Sunnyvale, California]) for one of the following viruses: adenovirus, endemic human coronavirus (HKU1, NL63, 229E, or OC43), human metapneumovirus, influenza A (H1N1pdm2009 or H3N2), influenza B, human parainfluenza virus (type 1, 2, 3, or 4), respiratory syncytial virus (RSV), rhinovirus/enterovirus (the latter are not distinguished on the panel), or SARS-CoV-2;

2. Symptomatic criterion: at least one new sign or symptom associated with a lower or upper respiratory infection following hospital admission, including conjunctivitis, cough, hypoxia, increased endotracheal secretions, increased ventilator settings, increased work of breathing, nasal congestion, rhinorrhea, sneezing, or tachypnea (fever was included if other sources of fever were ruled out);

3. Chronologic criterion: the onset of symptoms was on or after a minimum number of days from hospital admission for each specific virus listed below. Minimum intervals were defined as being one day longer than the upper limit of the incubation period for each virus described in the American Academy of Pediatrics Red Book^1^ (for viruses except SARS-CoV-2).

| Virus | **Minimum hospital day of symptom onset** |
| --- | --- |
| **Adenovirus** | 15 |
| **Coronavirus** | 6 |
| **Metapneumovirus** | 6 |
| **Influenza A** | 5 |
| **Influenza B** | 5 |
| **Parainfluenza** | 7 |
| **RSV** | 7 |
| **Rhino/enterovirus** | 8 |
| **SARS-CoV-2** | 11* |

*For SARS-CoV-2, The minimum hospital day of symptom onset for a definite HARVI was hospital day 15 from March 2020 to December 2021, then was changed to hospital day 11 after the emergence of the omicron variant characterized by a shorter incubation period.

For SARS-CoV-2, surveillance was also conducted for possible HARVIs, which were defined as above except that symptom onset was at an interval following admission between the minimum (2 days) and maximum incubation for the virus.

**HARVI surveillance**: At a regular interval, one infection preventionist runs a report in the electronic health record (EHR) for all positive respiratory virus PCR tests on hospitalized patients. Patients who were tested on a hospital day that was less than the maximum incubation period of that virus since admission are immediately excluded. The remaining cases have their records reviewed to determine if they were symptomatic and when symptoms started. The date of event is the earlier of the date of testing or the date of symptom onset. We use the table provided above to distinguish definite HARVIs vs POA (or possible HARVIs). We only include the definite HARVIs in our surveillance.

Statistical Process Control Chart

To analyze monthly HARVI data, a statistical process control u-chart (implemented in QIMacros®, KnowWare International, Inc., Denver, Colorado) was used that calculates control limits that vary by month depending on the size of the patient-days denominator.

Supplemental Results

| Supplementary Table 1. Viruses detected in healthcare-associated respiratory viral infections from January 2022 to June 2023 | | | | | | | | | | | |
| --- | --- | --- | --- | --- | --- | --- | --- | --- | --- | --- | --- |
|  | Number of HARVIs | | | | | | | | | | |
| Month | Total | ADV | ccCoV | hMPV | Flu A | Flu B | PIV | RSV | REV | SARS-CoV-2 | Co-infections |
| Jan-22 | 10 | 0 | 0 | 1 | 0 | 0 | 0 | 0 | 3 | 5 | 1 |
| Feb-22 | 6 | 0 | 0 | 0 | 0 | 0 | 0 | 0 | 6 | 0 | 0 |
| Mar-22 | 5 | 0 | 0 | 0 | 0 | 0 | 1 | 0 | 3 | 1 | 0 |
| Apr-22 | 6 | 1 | 1 | 0 | 0 | 0 | 0 | 0 | 4 | 0 | 0 |
| May-22 | 6 | 0 | 1 | 0 | 0 | 0 | 1 | 0 | 3 | 1 | 0 |
| Jun-22 | 5 | 0 | 0 | 0 | 0 | 0 | 0 | 0 | 5 | 0 | 0 |
| Jul-22 | 2 | 0 | 0 | 0 | 0 | 0 | 0 | 0 | 2 | 0 | 0 |
| Aug-22 | 6 | 1 | 0 | 0 | 0 | 0 | 0 | 0 | 5 | 0 | 0 |
| Sep-22 | 8 | 0 | 1 | 0 | 0 | 0 | 0 | 1 | 6 | 0 | 0 |
| Oct-22 | 6 | 1 | 0 | 0 | 0 | 0 | 0 | 1 | 4 | 0 | 0 |
| Nov-22 | 14 | 2 | 1 | 0 | 2 | 0 | 2 | 0 | 5 | 1 | 1 |
| Dec-22 | 22 | 1 | 0 | 0 | 2 | 0 | 4 | 2 | 7 | 3 | 3 |
| Jan-23 | 12 | 1 | 3 | 1 | 0 | 0 | 0 | 0 | 3 | 2 | 2 |
| Feb-23 | 14 | 1 | 2 | 0 | 0 | 0 | 1 | 1 | 7 | 1 | 1 |
| Mar-23 | 10 | 2 | 0 | 1 | 0 | 0 | 0 | 0 | 6 | 0 | 1 |
| Apr-23 | 8 | 1 | 0 | 0 | 0 | 0 | 2 | 0 | 2 | 1 | 2 |
| May-23 | 14 | 2 | 0 | 1 | 0 | 0 | 3 | 0 | 6 | 0 | 2 |
| Jun-23 | 8 | 1 | 0 | 0 | 0 | 0 | 1 | 0 | 5 | 0 | 1 |
| Mar-22 to Oct-22 | 44 | 3 | 3 | 0 | 0 | 0 | 1 | 2 | 32 | 2 | 0 |
| Nov-22 to Jun-23 | 102 | 11 | 6 | 3 | 4 | 0 | 13 | 3 | 41 | 8 | 13 |
| HARVI – healthcare-associated respiratory viral infections  ADV – respiratory adenoviruses  ccCoV – common cold coronaviruses (HKU1, NL63, 229E, or OC43)  hMPV – human metapneumovirus  Flu A – influenza A (H1N1pdm09 or H3N2)  Flu B – influenza B  PIV – parainfluenza virus (1, 2, 3, or 4)  RSV – respiratory syncytial virus  REV – rhinovirus or enterovirus  SARS-CoV-2 – severe acute respiratory syndrome coronavirus 2. Only includes definite healthcare-associated infections | | | | | | | | | | | |

| Supplementary Table 2. Length of stay prior to onset of symptoms in healthcare-associated respiratory viral infections | | | |
| --- | --- | --- | --- |
| Time period | No. HARVI with LOS data | Median LOS prior, days | IQR, days |
| Jan 19 – Feb 20 | 68 | 22 | 15-49.25 |
| Mar 20 – Oct 22 | 95 | 25 | 14-66 |
| Nov 22 – Jun 23 | 102 | 24.5 | 13-64.75 |

Additional respiratory virus test positivity figures

These figures include all respiratory virus testing at our local laboratory (HARVIs and community infections)

Supplementary Figure 1


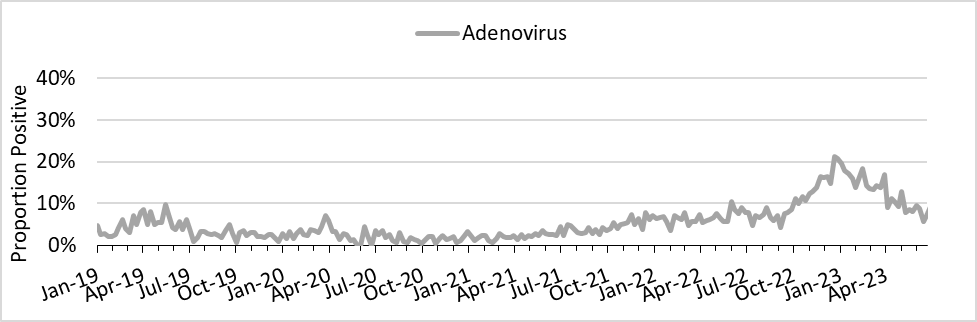


Supplementary Figure 2


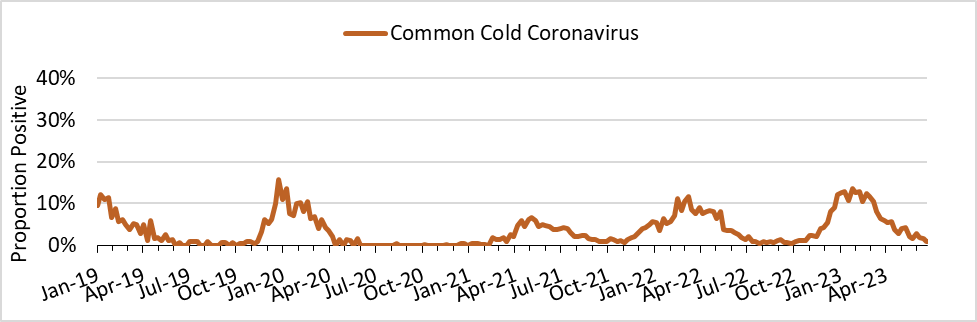


Supplementary Figure 3
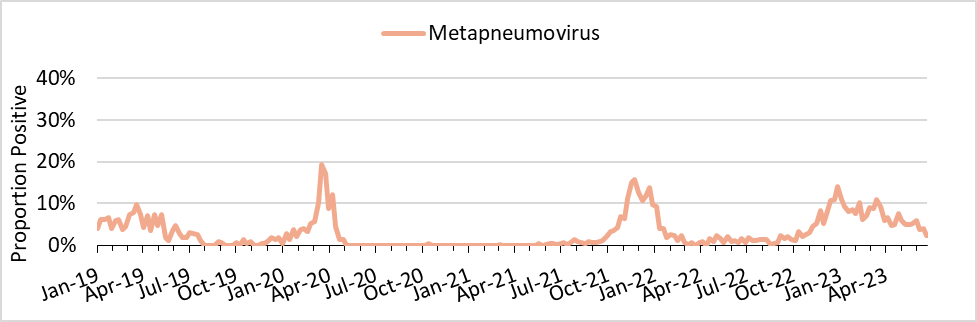


Supplementary Figure 4


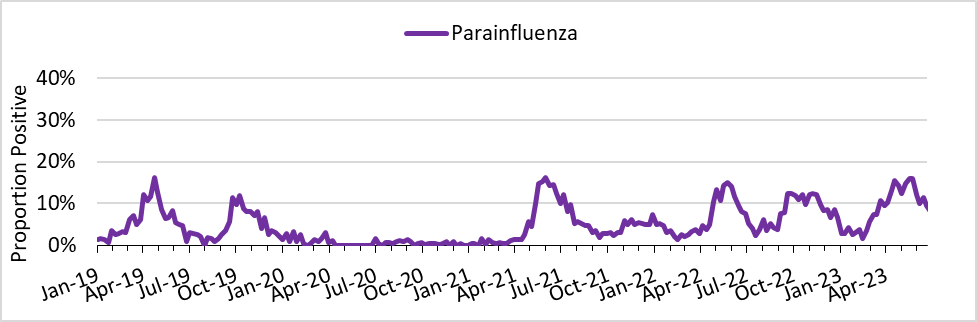


Supplementary Figure 5


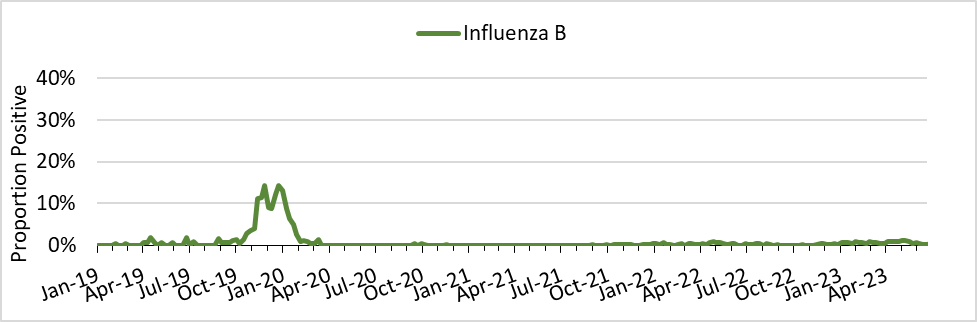

Supplement: Supplementary file 1 [file S0899823X23002003sup001.docx]
